# Reconsidering the generic status of *Dolichonobia* Meyer, 1974 with the description of a new species of *Mesobryobia* Wainstein, 1956 (Prostigmata, Tetranychidae) and a diagnostic key to world species

**DOI:** 10.3897/zookeys.1296.211177

**Published:** 2026-10-27

**Authors:** Nasreldeen Ahmed Elgoni, Jawwad Hassan Mirza, Fahad Jaber Alatawi

**Affiliations:** 1 Department of Plant Protection, College of Food and Agriculture Sciences, King Saud University, Riyadh, Saudi Arabia Department of Plant Protection, College of Food and Agriculture Sciences, King Saud University Riyadh Saudi Arabia

**Keywords:** Diagnosis, diversity, Mesobryobia (Mesobryobia) neobrevillosa, morphology, subgenera, taxonomy

## Abstract

The present research reevaluates the diagnostic characters of *Dolichonobia* Meyer and demotes it to a subgenus of *Mesobryobia*. Consequently, *Mesobryobia* is divided into two subgenera: Mesobryobia (Mesobryobia) and Mesobryobia (Dolichonobia), differentiated by the shape of the peritremes: anastomosed and simple, or bifurcate, respectively. Furthermore, a new species, Mesobryobia (Mesobryobia) neobrevillosa**sp. nov**., is described and illustrated based on females collected from grasses (Poaceae) in Jazan Province, Saudi Arabia. Additionally, a key to all species of the genus *Mesobryobia* is provided.

## Introduction

The tribe Hystrichonychini (Prostigmata, Tetranychidae, Bryobiinae) comprises 17 genera; among them, three genera, *Monoceronychus* McGregor, 1945, *Mesobryobia* Wainstein, 1956, and *Dolichonobia* Meyer, 1974, are morphologically related and can be differentiated from the other genera by their elongate body shape and development of the propodosomal lobes. *Monoceronychus* comprises 20 species and is unique among the three due to the presence of three anterior propodosomal lobes (Bolland et al. 1998).

The genus *Mesobryobia* comprises 11 described species, reported from Armenia, India, Kazakhstan, Pakistan, and South Africa (Migeon and Dorkeld 2026). Wainstein (1960) considered *Mesobryobia* to be a subgenus of *Monoceronychus*. Despite this, Tuttle and Baker (1968), Meyer (1974, 1987), Prasad (1975), Mitrofanov et al. (1987), and Kamran et al. (2021) continued to treat *Mesobryobia* at the genus level.

Meyer (1974) erected the genus *Dolichonobia* for two species: *D. prolata* Meyer, 1974 (type species) and *D. faceta* Meyer, 1974. Later, Cui and Tan (1993) described a third species, *D. altaiensis* Cui & Tan, 1993. *Dolichonobia* is morphologically related to *Mesobryobia* in having setae *f*_1_ and *h*_1_ on strong tubercles, but it differs in having poorly developed propodosomal lobes (Meyer 1974; Bolland et al. 1998).

The validity of *Dolichonobia* as a genus became doubtful after Kamran et al. (2021) argued that two *Mesobryobia* species, *M. delospermae* Meyer, 1974 and *M. brevillosa* Meyer, 1974, have extremely small (i.e. poorly developed) propodosomal lobes, which were considered a poor generic diagnostic character. Furthermore, *Mesobryobia* species were differentiated based on poorly or well-developed propodosomal lobes in the identification key (Kamran et al. 2021). The present study aimed to validate the taxonomic status of *Dolichonobia*, describe a new *Mesobryobia* species based on females, and develop a key to world species of *Mesobryobia*.

## Materials and methods

The diagnostic characters of two genera, *Dolichonobia* and *Mesobryobia*, and the described species were critically reviewed based on published taxonomic literature (Wainstein 1956; Bagdasarian 1959; Tuttle and Baker 1968; Meyer 1974, 1987; Prasad 1975; Mitrofanov et al. 1987; Cui and Tan 1993; Kamran et al. 2021). The specimens of the new *Mesobryobia* species were collected by shaking the plant’s vegetative parts over white sheets and preserved in small vials containing 70% ethanol. Specimens were examined and identified under a phase-contrast microscope (DM2500, Leica, Wetzlar, Germany). Different body parts were captured using an auto-montage software system (Syncroscopy, Cambridge, UK) attached to a phase-contrast microscope (DM2500, Leica, Germany). These images were used as templates and illustrated in Adobe Illustrator (Adobe Systems Inc., San Jose, CA, USA). All measurements are given in micrometers (μm). Length and width of the idiosoma were measured as the distance between setae *v*_2_–*h*_1_ and *c*_3_–*c*_3_, respectively. Setal lengths were measured from their basal insertions to their tips. Distances between setae were also measured as the distance between their insertions. Legs were measured from the trochanter to the pretarsus (bases of claws and empodium). The morphological terminology used follows Lindquist et al. (1985). The types of the new species have been deposited at the King Saud University Museum of Arthropods (**KSMA**, Acarology section), Department of Plant Protection, College of Food and Agriculture Sciences, King Saud University, Riyadh, Saudi Arabia.

## Results and discussion

**Figure 1.**
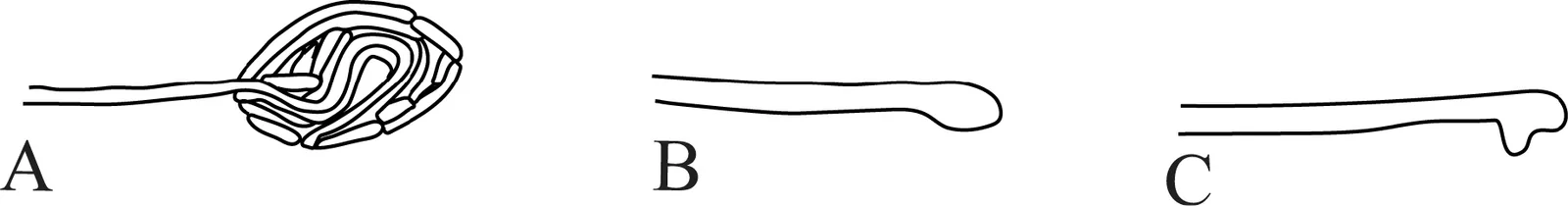
Shape of peritremes in female. **A**. Anastomose in Mesobryobia (M.) neobrevillosa sp. nov.; **B**. Simple bulb in M. (D.) faceta (Meyer, 1974) (redrawn from Meyer 1974); **C**. Bifurcate in M. (D.) altaiensis (Cui & Tan, 1993) (redrawn from Cui and Tan 1993).

The genus *Dolichonobia* is hereby proposed as a subgenus of *Mesobryobia*. Consequently, two subgenera are recognized in *Mesobryobia* based on differences in the peritremes shape: Mesobryobia (Mesobryobia) Wainstein with anastomosed peritremes (Fig. 1A), while Mesobryobia (Dolichonobia) Meyer with simple or bifurcate peritremes (Fig. 1B, C). The generic diagnosis of *Mesobryobia* is also updated. Furthermore, a new species, Mesobryobia (Mesobryobia) neobrevillosa sp. nov., is described based on a female, found inhabiting wild grasses (Poaceae), in the southern region of Saudi Arabia. Additionally, a key for all 15 species of the genus *Mesobryobia* is provided.

*Dolichonobia* was differentiated from *Mesobryobia* by the poorly developed propodosomal lobes (Meyer 1974; Bolland et al. 1998). Previously, Kamran et al. (2021) argued that propodosomal lobes development state was an invalid diagnostic character for generic differentiation of *Mesobryobia*. Additionally, two *Mesobryobia* species, *M. delospermae* and *M. brevillosa*, have been described with poorly developed propodosomal lobes. Consequently, the taxonomic boundary between the *Dolichonobia* and *Mesobryobia* became debatable. In the family Tetranychidae, different generic revisions have been reported related to the state of propodosomal lobe development or the character itself.

The state of the morphological character, being poorly/weakly or strongly developed, has previously been criticized in the two genera within the same tribe (Hystrichonychini) of *Dolichonobia* and *Mesobryobia*. Mahdavi et al. (2024) considered the feature of strong or small dorsal tubercles an inconsistent diagnostic character for distinguishing *Aplonobia* Womersley, 1940, and *Paraplonobia* Wainstein, 1960, respectively. Such character states heavily rely on the observer’s judgment and lack a clear definition (Mahdavi et al. 2024). In the current scenario, the state of propodosomal lobes between *Dolichonobia* and *Mesobryobia* poses a similar challenge, which has also been highlighted by Kamran et al. (2021), at least for *Mesobryobia*.

The propodosomal lobe character has been found variable between the genera in the other tribe Bryobinii. *Bryobia* Koch, 1836, and *Pseudobryobia* McGregor, 1950, were originally distinguished by with or without prominent propodosomal lobes, respectively (Bolland et al. 1998). Later, this state of propodosomal lobes was found to be variable among *Bryobia* species, and diagnoses of both genera were updated to rely only on the number of setae on coxae I–II (Arabuli et al. 2019).

The comprehensive analysis of all species of *Dolichonobia* and *Mesobryobia* in the current study revealed the shape of peritremes as a persistent morphological character. This character has been reported in the amended diagnosis of *Mesobryobia* by Kamran et al. (2021) as peritremes anastomosing present in all 11 of its species, while simple or bifurcate in three *Dolichonobia* species. Therefore, *Dolichonobia* could not be synonymized with *Mesobryobia*

The shape of peritremes could be a potential diagnostic character at the subgeneric level. Wainstein (1960) divided the genus *Petrobia* into three subgenera based on the shape of the peritremes: Petrobia (Petrobia) with anastomosing peritremes; Petrobia (Tetranychina) with simple, straight, or hooked peritremes; and Petrobia (Mesotetranychus) with simple, sometimes ending in a small fork. *Dolichonobia* holds strong morphological affinity with *Mesobryobia*, and it would be more than correct to consider it as a subgenus under *Mesobryobia*.

### Family Tetranychidae Donnadieu, 1875

**Subfamily Bryobiinae Berlese, 1913**

**Tribe Hystrichonychini Pritchard & Baker, 1955**

#### Genus Mesobryobia

Taxon classificationAnimaliaTrombidiformesTetranychidae

Wainstein, 1956

73D7B5E6-C5B2-507F-B33D-88B9C238D96F

##### Type species.

*Mesobryobia cervus* Wainstein, 1956: 73.

##### Updated diagnosis.

Median propodosomal lobe absent; propodosoma with three pairs of setae; opisthosoma with 10 pairs of setae; idiosoma elongate or oval, setae *f*_1_ more or less widely spaced than the first three pairs of dorsocentral setae (*c*_1_, *d*_1_, *e*_1_).

##### Remarks.

*Mesobryobia* can be distinguished from the related *Monoceronychus* (20 species) by the absence of a median propodosomal lobe (vs present).

#### Subgenus Mesobryobia

Taxon classificationAnimaliaTrombidiformesTetranychidae

Wainstein, 1956

2876C57D-AD1C-58A4-84F8-9E60D0810427

##### Type species.

*Mesobryobia cervus* Wainstein, 1956: 73.

##### Number of species included.

12 (including new species).

##### Diagnosis.

Peritremes anastomose distally.

#### Subgenus Dolichonobia

Taxon classificationAnimaliaTrombidiformesTetranychidae

Meyer, 1974

4AAD4D16-AC71-5D5B-AC80-C1EB358F04C2

##### Type species.

*Mesobryobia prolata* (Meyer, 1974): 64. By original designation.

##### Number of species included.

Three originally described in the genus.

##### Diagnosis.

Peritremes distally simple or bifurcate.

### New species

#### Mesobryobia neobrevillosa sp. nov.

Taxon classificationAnimaliaTrombidiformesTetranychidae

A5580F79-B94C-5352-A3F9-24256396D2BD

https://zoobank.org/EBE45457-B9B5-421D-8636-2AC8CF967A2A

Figs 2, 3, 4, 5

##### Type material.

***Holotype***: Saudi Arabia • ♀ Jazan; 17°11.533'N, 42°46.369'E; 7 Oct. 2025; E.M. Khan, N.A. Elgoni and H.M.S. Ali leg.; grasses (Poaceae); KSMAAS25-Tet-Mes-H. ***Paratypes***: • 2 ♀; same data as holotype; KSMAAS25-Tet-Mes-P1-2.

##### Diagnosis.

(based on adult female) Opisthosomal plate absent; femora III–IV each with two tactile setae; genua I–IV with 3–3–2–3 tactile setae; tibiae III–IV each with three tactile setae.

##### Description.

**Female** (*n* = 3). Length of idiosoma (from setae *v*_2_–*h*_1_) (excluding gnathosoma) 410 (418–421) (including gnathosoma) 459 (455–471), maximum width at level of setae *c*_3_ series 191 (195–200).

**Figure 2.**
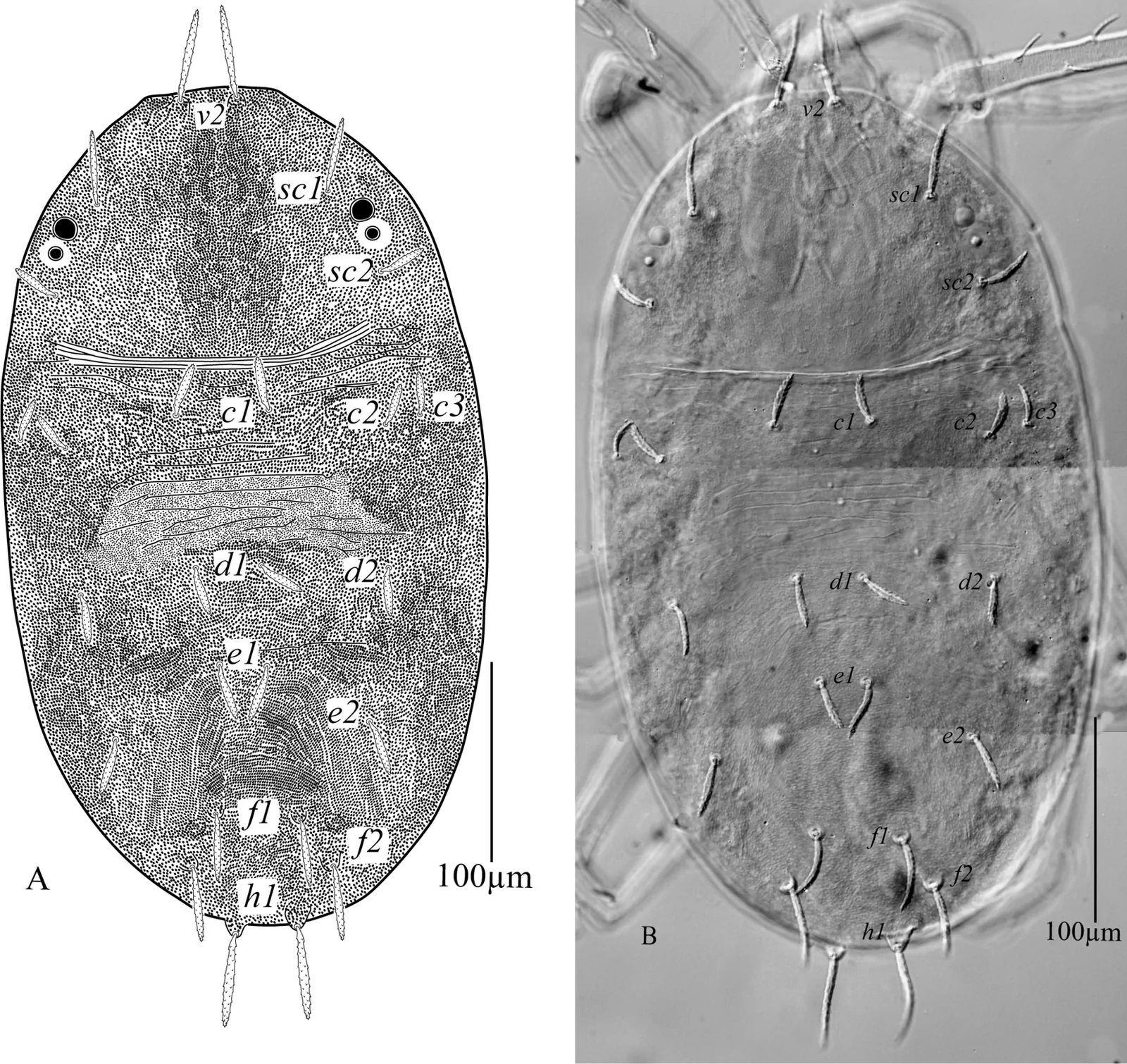
Mesobryobia (M.) neobrevillosa sp. nov., female. **A**. Dorsum; **B**. Microphotograph of dorsum.

***Dorsum*** (Fig. 2A, B). Anterior propodosomal lobes absent; instead propodosomal setae *v*_2_ present on prominent tubercles. Propodosoma and hysterosoma densely granulated, area anterior to setae *d*_1_ with few widely spaced, transverse striae and lightly granulated; granulation between setae *e*_1_, *e*_2_ and *f*_1_ forming an inverted U shape, medially with curved, transverse granulation. All dorsal setae lanceolate, serrate, setae *f*_1_, *f*_2_ and *h*_1_ inserted on strong/prominent tubercles. Lengths of dorsal setae: *v*_2_ 48 (48–50), *sc*_1_ 38 (38–41), *sc*_2_ 28 (28–30), *c*_1_ 30 (25–29), *c*_2_ 25 (21–23), *c*_3_ 20 (19–21), *d*_1_ 25 (25–21), *d*_2_ 26 (20–25), *e*_1_ 26 (25–26), *e*_2_ 28 (24–28), *f*_1_ 22 (21–23), *f*_2_ 36 (23–36), *h*_1_ 49 (48–50); distances between dorsal setae: *v*_2_–*v*_2_ 21 (18–24), *sc*_1_–*sc*_1_ 113 (111–115), *sc*_2_–*sc*_2_ 160 (157–159), *c*_1_–*c*_1_ 41 (39–43), *c*_2_–*c*_2_ 157 (161–162), *d*_1_–*d*_1_ 31 (31–35), *d*_2_–*d*_2_ 154 (149–156), *e*_1_–*e*_1_ 20 (16–20), *e*_2_–*e*_2_ 125 (121–126), *f*_1_–*f*_1_ 41 (37–39), *f*_2_–*f*_2_ 66 (68–70), *h*_1_–*h*_1_ 26 (23–25), *v*_2_–*sc*_1_ 61 (59–61), *sc*_1_–*sc*_2_ 46 (42–46), *sc*_2_–*c*_3_ 67 (63–77), *sc*_2_–*c*_2_ 71 (71–74), *sc*_2_–*c*_1_ 82 (80–85), *c*_1_–*c*_2_ 54 (53–62), *c*_2_–*c*_3_ 16 (16–18), *c*_1_–*d*_1_ 74 (73–75), *c*_2_–*d*_2_ 67 (67–69), *d*_1_–*e*_1_ 48 (47–51), *d*_2_–*e*_2_ 75 (71–76), *e*_1_–*f*_1_ 72 (62–70), *e*_2_–*f*_2_ 71 (61–71), *f*_1_–*f*_2_ 25 (25–31), *f*_1_–*h*_1_ 49 (36–38), *f*_2_–*h*_1_ 31 (30–36). Setae *f*_1_ normal in position, distance between *f*_1_–*f*_1_ sub equal spaced with *c*_1_–*c*_1_.

**Figure 3.**
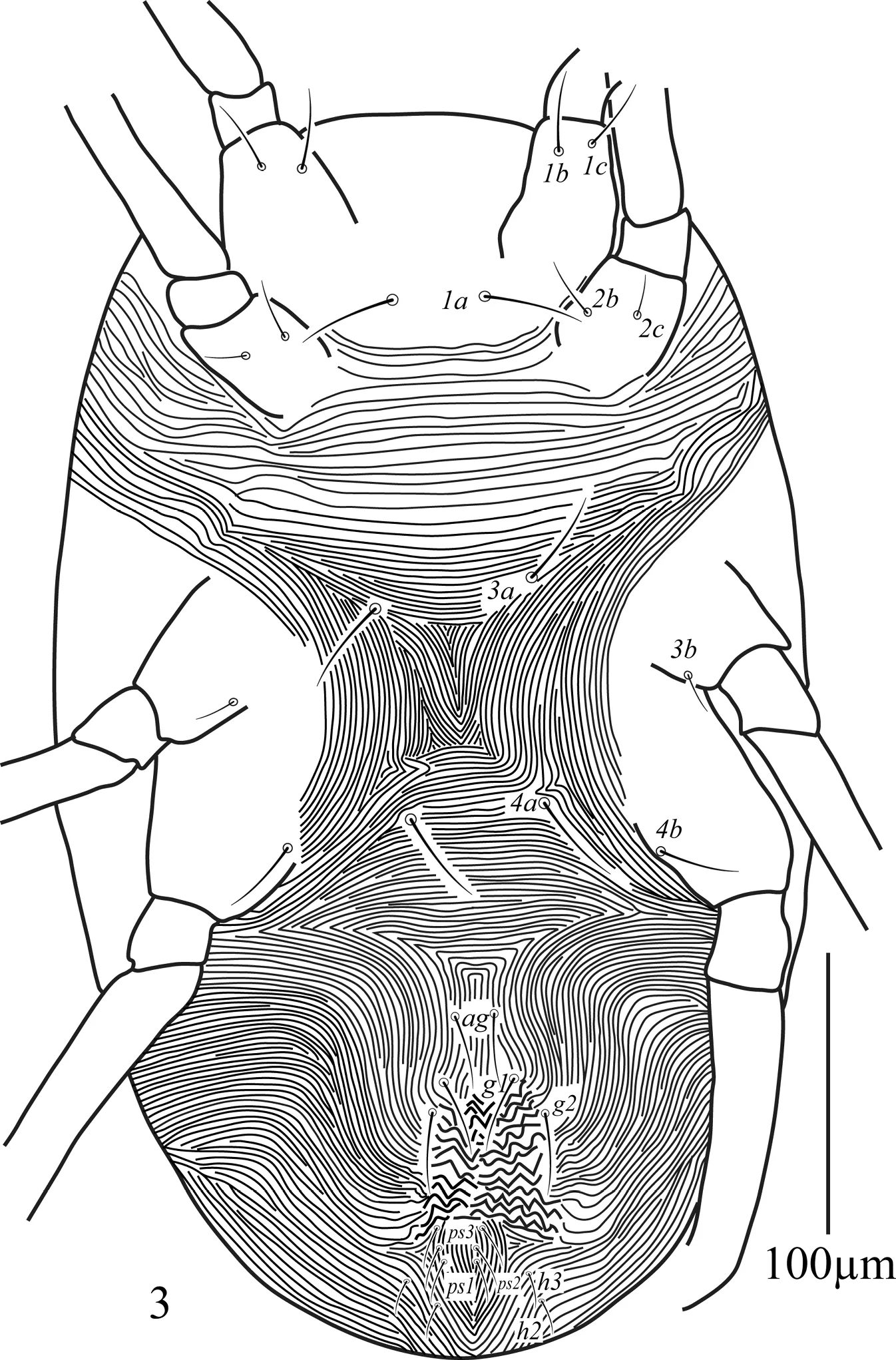
Mesobryobia (M.) neobrevillosa sp. nov., female, venter. Scale bar: 100 μm.

***Venter*** (Fig. 3). Idiosoma ventrally with widely spaced transverse striae from anterior level of coxae II to 3*a*, area between coxae III and IV with curved longitudinal striae medially forming a V shape, area posterior to setae *4*a with transverse striae and around setae *ag* with longitudinal striae; venter striations simple, smooth. Lengths of ventral setae: 1*a* 36 (34–37), 3*a* 34 (29–34), 4*a* 34 (33–35), 1*b* 26 (27–30), 1*c* 25 (22–26), 2*b* 23 (23–25), 2*c* 16 (12–15), 3*b* 15 (14–16), 4*b* 28 (25–28); distances between inter–coxal and coxal setae: 1*a*–1*a* 26 (21–26), 1*b*–1*c* 10 (8–11), 2*b*–2*c* 13 (13–15), 3*a*–3*a* 46 (41–45), 4*a*–4*a* 39 (36–39); aggenital setae: *ag* 25 (22–26), *ag*–*ag* 11 (9–12); genital setae two pairs: *g*_1_ 26 (25–30), *g*_2_ 28 (27–29), *g*_1_–*g*_1_ 21 (18–22), *g*_2_–*g*_2_ 38 (37–41); three pairs of pseudanal setae (anal setae): *ps*_1_ 14 (13–15), *ps*_2_ 14 (14–18), *ps*_3_ 15 (15–16); distances between pseudanal setae, *ps*_1_–*ps*_2_ 6 (7–10), *ps*_2_–*ps*_3_ 8 (8–11), *ps*_1_–*ps*_1_ 7 (7–9), *ps*_2_–*ps*_2_ 6 (7–8), *ps*_3_–*ps*_3_ 33 (31–32); ventrocaudal (para anal) setae two pairs: *h*_2_ 11 (9–11), *h*_3_ 12 (11–13), distances between ventrocaudal setae, *h*_2_–*h*_2_ 32 (32–36), *h*_3_–*h*_3_ 38 (38–41). Spermatheca not visible.

**Figure 4.**
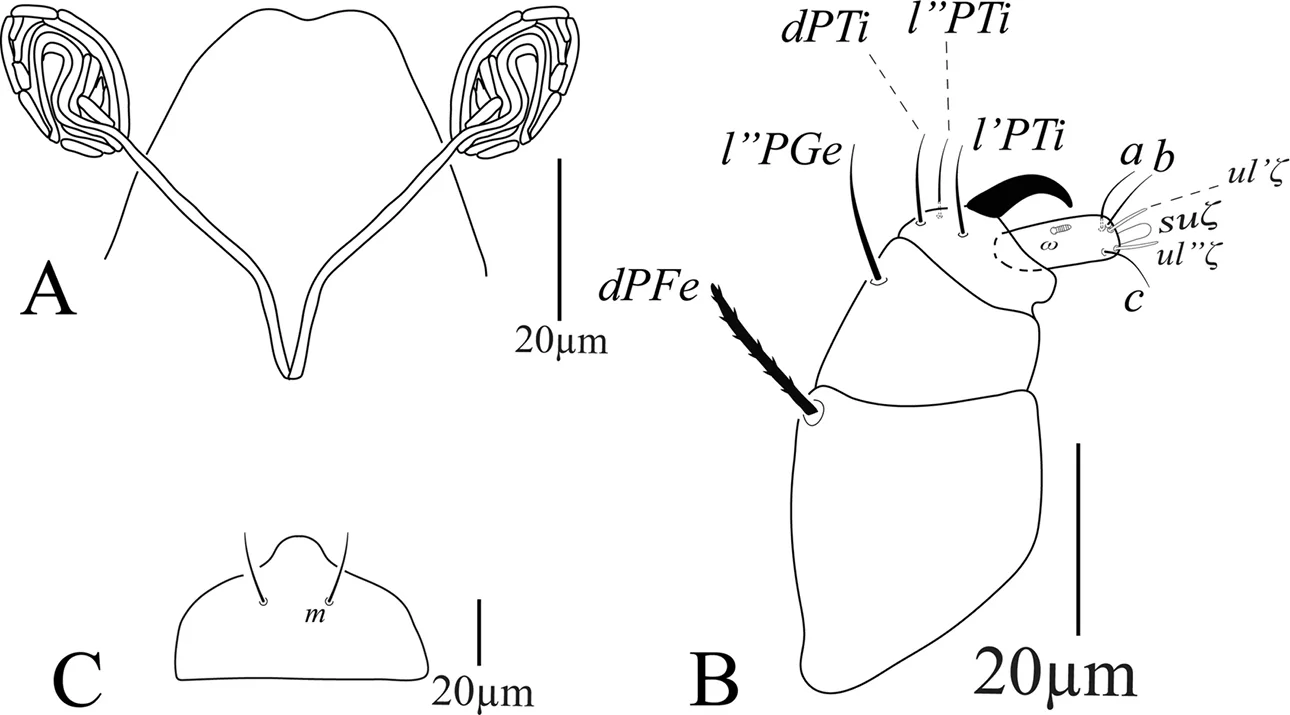
Mesobryobia (M.) neobrevillosa sp. nov., female. **A**. Stylophore and peritremes; **B**. Palp; **C**. Subcapitular.

***Gnathosoma*** (Fig. 4A–C). Stylophore slightly incised anteriorly. Peritremes ending with anastomosing chambers (Fig. 4A). Palp (Fig. 4B) palp femur with one setae *d* 16 (15–18) and genu with one seta *l”* 15 (14–16); palp tibia with three setae, *d* 10 (8–10), *l”* 10 (8–11), *l’* 9 (9–10) and a palp tibial claw; palptarsus with three simple setae *a* 7 (7–9), *b* 7 (7–8), *c* 6 (6–8); three eupathidia *suζ* 6 (6), *ul’’ζ* 5 (5–6), *ul’ζ* 5 (4–5), solenidion *ω* 3 (2–3) long. Subcapitular (Fig. 4C). setae *m* 23 (21–24), *m*–*m* 18 (16–19).

**Figure 5.**
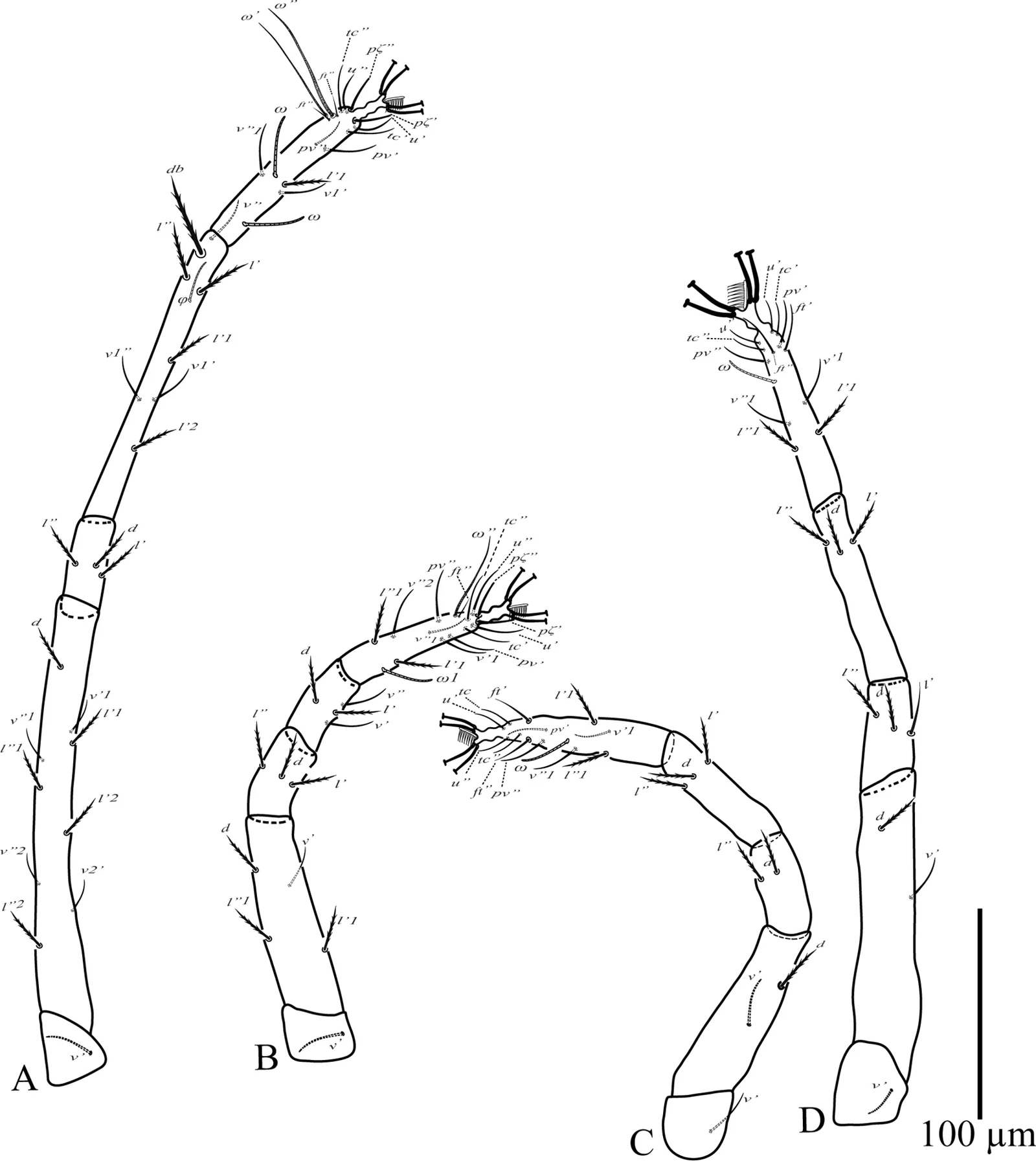
Mesobryobia (M.) neobrevillosa sp. nov., female, **A**. Leg I; **B**. Leg II; **C** Leg III; **D**. Leg IV.

***Legs*** (Fig. 5A–D). Length of legs I–IV (without coxae): 418 (397–409), 212 (212–215), 235 (225–240), 303 (295–304), respectively; leg I: trochanter 25 (18–25), femur 161 (153–162), genu 39 (30–41), tibia 120 (119–123), tarsus 77 (73–77); leg II: trochanter 23 (15–23), femur 79 (72–80), genu 31 (25–30), tibia 36 (33–38), tarsus 52 (48–51); leg III: trochanter 25 (21–26), femur 69 (54–67), genu 26 (24–28), tibia 48 (43–48), tarsus 54 (52–55); leg IV: trochanter 33 (25–34), femur 98 (93–98), genu 43 (37–44), tibia 72 (67–73), tarsus 72 (69–72); leg setal counts on I–IV (solenidia and eupathidia are in parenthesis): coxae 2–2–1–1, trochanters 1–1–1–1; femora 9–4–2–2; genua 3–3–2–3; tibiae 8(1*φ*)–4–3–3; tarsi 9 (2*pζ*+2*ω*)+2 dupl.–11 (2*pζ*+1*ω*)+1 dup.–12(1*ω*)–12(1*ω*). Empodial pad provided with approximately 8 pairs of tenent hairs.

##### Males and immature stages.

Not found.

##### Etymology.

The species name is derived from the closely related species Mesobryobia (M.) brevillosa Meyer.

##### Remarks.

The new species is closely related to *M. brevillosa* Meyer in having setae *v*_2_ present on prominent tubercles, the peritremes distally anastomose, and femur I with nine setae. However, the new species distinguished from *M. brevillosa* by the absence of the opisthosomal plate (vs present and including setae *f*_2_, *h*_1_); femora III–IV each with two tactile setae (vs each with four tactile setae); genua I–IV with 3–3–2–3 tactile setae (vs 5–5–4–6 tactile setae); tibiae III–IV each with three tactile setae (vs each with seven tactile setae); and first and second pairs of propodosomal setae *v*_2_ and *sc*_1_ subequal in length (vs setae *v*_2_ about twice length of *sc*_1_ in *M. brevillosa*).

### Key to the subgenera and all species of *Mesobryobia*

The species M. (M.) delospermae not included in this key because its described based on the male and deutonymph.

**Table:** 

1	Peritremes anastomose distally	***Mesobryobia* subgenus [2]**
–	Peritremes simple or bifurcate distally	***Dolichonobia* subgenus [12]**
2	Anterior lobes very small or absent	**3**
–	Well-developed, anterior lobes over rostrum present	**4**
3	Opisthosomal plate include setae *f*_2_, h1; genua I–IV with 5–5–4–6 tactile setae	***M. brevillosa* Meyer, 1974**
–	Opisthosomal plate absent; genua I–IV with 3–3–2–3 tactile setae	***M. neobrevillosa* sp. nov**.
4	Most dorsal body setae lanceolate, but some distinctly different in form	**5**
–	All dorsal body setae orbicular, obovate, spatulate, or subspatulate	**8**
5	Setae *f*_2_ lanceolate	**6**
–	Setae *f*_2_ subspatulate or obovate	**7**
6	Setae *h*_1_ almost as long as setae *v*_2_; setae *f*_2_ set on small tubercles, narrowly lanceolate and as long as *f*_1_	***M. comatula* Meyer, 1974**
–	Setae *h*_1_ almost twice the length of setae *v*_2_; setae *f*_2_ set on prominent tubercles, broadly lanceolate or subspatulate and distinctly longer than *f*_1_	***M. genisi* Meyer, 1974**
7	Setae *sc*_1_ and *h*_1_ subspatulate	***M. jobneri* Prasad, 1975**
–	Setae *sc*_1_ lanceolate, setae *h*_1_ obovate	***M. parmata* Meyer, 1974**
8	Dorsal body setae orbicular	**9**
–	Dorsal body setae subspatulate, spatulate or obovate	**11**
9	Setae *f*_2_ and *h*_1_ set on strong tubercles	***M. terpoghossiani* (Bagdasarian, 1959)**
–	Setae *f*_2_ and *h*_1_ not set on tubercles	**10**
10	Femur III and genua I–IV each with three tactile setae; prodorsal lobes equal in length than accompanying first pair of prodorsal setae *v*_2_	***M. punjabensis* Kamran, Khan & Alatawi, 2021**
–	Femur III with two tactile setae, genua I–IV each with four setae; prodorsal lobes longer than accompanying first pair of prodorsal setae *v*_2_	***M. keetchi* Meyer, 1987**
11	Femora I–II with 9–5 tactile setae respectively; genua I–IV each with four tactile setae	***M. foliata* Meyer, 1974**
–	Femora I–II with 8–6 tactile setae and genua I–IV with 2–2–3–3 tactile setae	***M. cervus* Wainstein, 1956**
12	Femora I–IV each with 6–3–2–2 tactile setae	***D. faceta* (Meyer, 1974)**
–	Femora I–IV each with different formula	**13**
13	Tibiae II–IV each with nine tactile setae; genua III–IV each with five tactile setae	***D. prolata* (Meyer, 1974)**
–	Tibiae II–IV with 6–4–6 tactile setae; genua III–IV each with four tactile setae	***D. altaiensis* (Cui & Tan, 1993)**

## Supplementary Material

XML Treatment for Mesobryobia

XML Treatment for Mesobryobia

XML Treatment for Dolichonobia

XML Treatment for Mesobryobia neobrevillosa

## Additional information

### Artificial Intelligence (AI) use

The authors accept full responsibility for the content of the manuscript, including the disclosure of any use of AI.

No AI tools were used in the preparation of this manuscript.

### Author ORCIDs

N.A. Elgoni https://orcid.org/0009-0007-7698-1377

J.H. Mirza https://orcid.org/0000-0002-1726-4331

F.J. Alatawi https://orcid.org/0000-0002-6824-2650

### Data availability

All of the data that support the findings of this study are available in the main text.
